# Birth outcomes across the spectrum of maternal age: dissecting aging effect versus confounding by social and medical determinants

**DOI:** 10.1186/s12884-021-04077-w

**Published:** 2021-09-01

**Authors:** Bolanle Olapeju, Xiumei Hong, Guoying Wang, Amber Summers, Irina Burd, Tina L. Cheng, Xiaobin Wang

**Affiliations:** 1 Department of Population, Family and Reproductive Health, Johns Hopkins University Bloomberg School of Public Health, 615 N. Wolfe Street, E4132, Baltimore, MD 21205-2179 USA; 2 Center for Communication Programs, Johns Hopkins Bloomberg School of Public Health, Baltimore, MD USA; 3 Integrated Research Center for Fetal Medicine, Department of Gynecology and Obstetrics, Johns Hopkins University School of Medicine, Baltimore, MD USA; 4 Department of Pediatrics, Cincinnati Children’s Hospital Medical Center, University of Cincinnati, Cincinnati, OH USA; 5 Department of Pediatrics, Johns Hopkins University School of Medicine, Baltimore, MD USA

**Keywords:** Age, Cesarean section, High-risk, Low birth weight, Minority, Preterm birth, Race

## Abstract

**Background:**

Given the trend of increasing maternal age and associated adverse reproductive outcomes in the US, this study aimed to assess whether this association is due to an independent aging or confounded by sociodemographic, biomedical, or behavioral determinants in a predominantly Black US population.

**Methods:**

Data was from 8509 women enrolled in the Boston Birth Cohort. Adverse reproductive outcomes included spontaneous preterm delivery, cesarean delivery, and low birth weight. Covariates included sociodemographic (parity, race/ethnicity, education, marital status, income, receipt of public assistance, nativity); biomedical (obesity, hypertensive disorders, diabetes mellitus); and behavioral (consistent intake of multivitamin supplements, support from father of baby, support from family, major stress in pregnancy, cigarette smoking, alcohol intake). Analysis included Lowess and marginal probability plots, crude and adjusted sequential logistic regression models to examine age-outcome associations and to what degree the association can be explained by the above covariables.

**Result:**

Overall, the study sample had high levels of spontaneous preterm birth (18%), cesarean delivery (33%) and low birth weight (26%). Unadjusted models showed no significant difference odds of spontaneous preterm birth by maternal age but higher odds of cesarean section (aOR: 1.77, 95% CI: 1.60, 1.95) and low birth weight (aOR: 1.15, 95% CI: 1.04, 1.28) among women 30 years or older. Adjustment for sociodemographic factors, biomedical conditions and behavioral factors revealed higher odds of spontaneous preterm birth: (aOR: 1.30, 95% CI: 1.14, 1.49), cesarean section deliveries (aOR: 1.68, 95% CI: 1.51, 1.87) and low birth weight (aOR: 1.36, 95% CI: 1.21, 1.53). Across all ages, optimal BMI status and consistent multivitamin supplement intake were protective of spontaneous preterm birth and low birth weight.

**Conclusion:**

In this high-risk minority population, we demonstrated that the association between increasing maternal age and adverse pregnancy outcomes was due to an independent aging effect and the presence of confounding by sociodemographic, biomedical, and behavioral factors. Some modifiable risk factors to counteract aging effect, include optimizing BMI and consistent intake of multivitamin supplement. A fundamental change in how care is provided to women, particularly low income Black women, is needed with emphasis on the protective role of optimal nutritional status.

**Trial registration:**

ClinicalTrials.gov Identifier: NCT03228875

**Supplementary Information:**

The online version contains supplementary material available at 10.1186/s12884-021-04077-w.

## Background

The mean age of mothers in the US is currently at a record high of 29 years, ranging from 27.1 years in American Indian or Alaska Native (AI/AN) women to 31.8 years in Asian women [1, 2]. The rising age of mothers has been attributed to increased birth rates among older women as provisional birth rates for 2019 show a decline women aged 15–34 and an increase for women aged 40–44 [3]. While delayed childbearing offers associated societal benefits such as improved health and education outcomes in children as well as financially and emotionally empowered women who are often more prepared for child rearing [4], it is also of clinical and public health importance as aging is associated with fertility decline, with the fecundity of women decreasing gradually at age 32 years and more rapidly after age 37 years [5]. Maternal age also influences the total number of births a woman has over a lifetime, impacting the composition and growth of the US population [6]. In addition, increased maternal age is associated with adverse pregnancy outcomes such as birth defects, preterm birth, cesarean delivery, preeclampsia, postpartum hemorrhage, gestational diabetes among others [7, 8].

The mechanisms through which increasing maternal age result in adverse pregnancy outcomes are not yet fully understood. In addition to fertility decline, aging also results in a worsened overall health of women [9, 10] including medical conditions such as hypertension, diabetes [11] and obesity [12], potentially confounding the relationship between maternal age and adverse pregnancy outcomes. Another major confounder is race/ethnicity as adverse pregnancy outcomes are more common among minority populations. Non-Hispanic Black and AI/AN women are two to three times more likely to die from pregnancy-related causes than non-Hispanic White women [13]. This race/ethnic disparity in pregnancy-related deaths has persisted over time and has recently been shown to worsen with the age of the woman [13]. Other potential confounders identified in the literature include psychosocial factors such as rural residence [14], low maternal socio-economic status [15], lower levels of education [14, 16] and inadequate prenatal care [7, 17–22].

Further research is needed to understand what extent the relationship between maternal age and adverse pregnancy outcomes is due to an aging effect or confounded by sociodemographic, biomedical, or behavioral factors. The complexity of these factors requires an analysis which is well-designed to interrogate how and to what degree each individual component, as well as the combination thereof, may ultimately contribute to pregnancy outcomes. Such research findings can inform existing guidelines and tailor interventions across the female life course, including the preconception, pregnancy, postpartum and interconception periods. In addition, such research would also inform whether existing guidelines and strategy to address adverse outcomes need to be further stratified by other factors (such as race/ethnicity) in order to mitigate long-lasting unfavorable trends in pregnancy outcomes.

Our study explores the relationship between maternal age and adverse outcomes- spontaneous preterm birth, cesarean delivery, and low birth weight - among a low-income minority US population. We explore to what extent the relationship between age and adverse outcomes is confounded by sociodemographic, biomedical, and behavioral determinants. Of particular interest is the role of modifiable risk factors such as pre-pregnancy body mass index (BMI) and multivitamin supplement intake during pregnancy as confounders. Our study findings aim to inform the design of contextually relevant approaches and interventions for similar low income minority US populations.

## Methods

### Study design

Study data are from the Boston Birth Cohort (BBC) study, which has been described elsewhere [23]. In summary, participants in the BBC study are women with a singleton, live infant (without major birth defects) enrolled from 1998 to date at the Boston Medical Center (BMC), a large urban hospital serving a predominantly Black, low-income, inner-city patient population. The BBC oversampled for preterm births and/or low birth weight in a 1:2 ratio compared with term, normal weight births. Exclusion criteria were deliveries with major birth defects or congenital chromosomal abnormalities, or due to multiple gestation or in-vitro fertilization. Among eligible mothers approached by the study team, enrollment rate was about 83%. This cross-sectional study analysis focuses on all women (*N* = 8509) enrolled in the BBC- a reflection of the patient population of the BMC. Data sources include a postpartum questionnaire administered by trained data collectors within 72 h of birth as well as participants’ medical records. The study protocol was approved by the Institutional Review Boards of Boston University Medical Center and Johns Hopkins Bloomberg School of Public Health.

### Study variables

The independent variable, maternal age was based on age at delivery from medical records categorized into decades as follows: less than 20; 20–29; 30–39; and 40 or more years. Data were extracted from medical records for the following dependent variables: i) spontaneous preterm delivery, defined as a live birth before 37 completed weeks of gestation with a clinical presentation of uterine contraction or rupture of membranes (yes or no); ii) cesarean delivery (yes or no); and iii) low birth weight defined as a birthweight less than 2500 g (yes or no).

Biomedical factors were also extracted from participants’ medical records. These included pregestational/ gestational diabetes (yes versus no) and hypertensive disorders defined as (yes versus no) the presence of any of the following diagnoses: gestational hypertension, preeclampsia, eclampsia, or hemolysis, elevated liver enzymes, and/or low platelets (HELLP) syndrome. In addition, year of delivery (< 2006 (the median) versus ≥2006) was included as a contextual variable.

Maternal self-reported variables assessed from the post-partum questionnaire included maternal race categorized into: Black (Black, African American, Cape Verdean or Haitian)) versus non-Black, parity (nulliparous versus multiparous), pre-pregnancy BMI from their weight and height (underweight, normal weight, overweight and obese), education (<College versus ≥College), marital status (unmarried versus married), year of delivery, receipt of public assistance including: WIC (Women Infants and Children), Food Stamps, AFDC (Aid to Families with Dependent Children), Housing assistance or Fuel assistance (yes versus no); yearly income (<$30,000 (the median), ≥$30,000 and unknown) and nativity (US born versus non-US born).

Behavioral factors were also self-reported and included: perceived support during pregnancy from the father of the baby (none or little, some, excellent, further dichotomized to excellent versus not excellent); perceived support during pregnancy from family members (none or little, some, excellent, further dichotomized to excellent versus not excellent); perceived stress in pregnancy -(yes versus no); cigarette smoking (never versus any), alcohol consumption (never versus any) and consistent multivitamin supplement (at least three times a week) throughout pregnancy (yes or no). Women were asked how often they took multivitamin or over the counter supplements in each trimester. Options included none, less than once a week, one to two times a week, three to five times a week and more than five times a week. Multivitamin supplement intake at least three times has been associated with improved pregnancy outcomes in this study population as documented previously [23].

### Statistical analysis

All analyses were conducted using STATA version 15 (College Station, TX: StataCorp LP). Chi square tests of association explored maternal characteristics across the different age groups. LOWESS (locally weighted scatterplot smoothing) plots were used to graph the distribution of adverse pregnancy outcomes, obesity, and multivitamin use in pregnancy by maternal age as well as race/ethnicity to highlight any racial differences in the relationship between these adverse outcomes and age. The relationship between maternal age and adverse outcomes were further explored using bivariate and sequential multivariable logistic regression models including the groups of sociodemographic, biomedical, and behavioral variables described above. Additional regressions were done among the subgroup of Black women only, given their higher risk of these outcomes. Finally, marginal probabilities of all adverse outcomes were plotted by maternal age. Missing data was minimal (< 8%) for all variables except yearly income (11%) and multivitamin supplement intake (12%). Initial description of the study population accounted for missing data as a separate category while subsequent analyses reclassified missing data using simple imputation (assigned to the race- and age- specific most frequent category). All *P*-values in the analyses were based on two-sided statistical tests and the Type I error rate was set at 0.05.

## Results

Table 1 presents the characteristics of the study population by maternal age. Overall, women were mostly Black, multiparous, had less than a college education, unmarried, on public assistance, reported an income of <$30,000. On average, about a fifth (19%) of women were obese, 13% had hypertensive disorders of pregnancy while 10% had diabetes mellitus. Less than half of all women reported excellent support from the father of their baby or from their families while 22% of women noted a very stressful pregnancy. Few women reported ever smoking cigarettes (19%), drinking alcohol (9%) or inconsistent vitamin supplement intake (23%). Of note, most maternal characteristics varied by age groups except drinking of alcohol and receipt of public assistance.

**Table 1:** Characteristics of the study population by maternal age groups (*N* = 8509)

Maternal Characteristics	< 20 years		20–29 years		30–39 years		40+ years		Total		***P***-value^**a**^
	N	%	N	%	N	%	N	%	N	%	
Total	907	100	4305	100	2960	100	337	100	8509	100	
**Sociodemographic**											
*Race/ethnicity*											< 0.001
Black^b^	488	53.8	2073	48.2	1613	54.5	219	65.0	4393	51.6	
Non-Black	419	46.2	2232	51.8	1347	45.5	118	35.0	4116	48.4	
*Parity*											< 0.001
Nulliparous	760	83.8	2103	48.9	751	25.4	49	14.5	3663	43.0	
Multiparous	147	16.2	2202	51.1	2209	74.6	288	85.5	4846	57.0	
*Education (n = 8349)*											< 0.001
< College	813	91.3	2733	64.9	1658	56.9	219	66.0	5423	65.0	
≥ College	77	8.7	1479	35.1	1257	43.1	113	34.0	2926	35.0	
*Marital Status (n = 8340)*											< 0.001
Not married	842	94.8	2984	70.8	1473	50.7	170	51.5	5469	65.6	
Married	46	5.2	1232	29.2	1433	49.3	160	48.5	2871	34.4	
*Public assistance*^*c*^ *(n = 8378)*											0.580
No	157	17.5	813	19.2	572	19.6	63	18.9	1605	19.2	
Yes	738	82.5	3423	80.8	2341	80.4	271	81.1	6773	80.8	
*Yearly Income in US$ (n = 7616)*											< 0.001
≥ $30,000	18	2.3	470	12.1	547	20.7	59	19.2	1094	14.4	
< $30,000	321	40.1	1861	48.1	1167	44.2	138	44.8	3487	45.8	
Don’t know	461	57.6	1538	39.8	925	35.1	111	36.0	3035	39.9	
*Place of birth (n = 8310)*											< 0.001
Not US born	309	34.7	2400	57.0	2096	72.8	270	81.3	5075	61.1	
US born	581	65.3	1809	43.0	783	27.2	62	18.7	3235	38.9	
**Biomedical**											
*Body Mass Index (n = 7930)*											< 0.001
< 19	97	11.3	324	8.1	109	3.9	6	1.9	536	6.8	
19–24	467	54.4	1899	47.5	1147	41.5	118	37.9	3631	45.8	
25–29	197	22.9	1003	25.1	853	30.9	103	33.1	2156	27.2	
≥ 30	98	11.4	770	19.3	655	23.7	84	27.0	1607	20.3	
*Hypertensive disorders of pregnancy (n = 8498)*											< 0.001
No	810	89.3	3830	89.1	2519	85.2	272	80.7	7431	87.4	
Yes	97	10.7	467	10.9	438	14.8	65	19.3	1067	12.6	
*Pregestational/gestational diabetes (n = 8500)*											< 0.001
No	876	96.7	3957	92.1	2524	85.3	260	77.2	7617	89.6	
Yes	30	3.3	340	7.9	436	14.7	77	22.8	883	10.4	
***Behavioral***											
*Father of baby support (n = 7829)*											< 0.001
None/Little	201	24.3	634	16.1	383	14.0	55	17.6	1273	16.3	
Some	278	33.6	1474	37.3	993	36.3	126	40.4	2871	36.7	
Excellent	349	42.1	1842	46.6	1363	49.8	131	42.0	3685	47.1	
*Family support (n = 7917)*											0.014
None/Little	47	5.6	301	7.5	215	7.7	30	9.3	593	7.5	
Some	368	44.2	1786	44.8	1225	44.1	167	51.9	3546	44.8	
Excellent	418	50.2	1900	47.7	1335	48.1	125	38.8	3778	47.7	
*Major stressful events in pregnancy*											0.030
No	663	75.8	3136	75.5	2262	79.1	256	79.3	6317	76.9	
Yes	212	24.2	1017	24.5	598	20.9	67	20.7	1894	23.1	
*Smoking cigarettes (n = 8441)*											< 0.001
Never	717	79.3	3321	77.8	2478	84.4	289	87.0	6805	80.6	
Ever	187	20.7	949	22.2	457	15.6	43	13.0	1636	19.4	
*Drinking alcohol (n = 8201)*											0.606
Never	798	91.3	3747	90.6	2630	91.5	290	90.6	7465	91.0	
Ever	76	8.7	387	9.4	243	8.5	30	9.4	736	9.0	
*Multivitamin supplement intake at least 3 times a week throughout pregnancy (n = 7496)*											< 0.001
No	273	34.3	1050	27.7	587	22.4	71	24.5	1981	26.4	
Yes	524	65.7	2742	72.3	2030	77.6	219	75.5	5515	73.6	

^a^*p*-value from chi square tests of association; ^b^ Maternal self-report as Black, African American, Haitian or Cape Verdian;^c^ receipt of public assistance including: WIC (Women Infants and Children), Food Stamps, AFDC (Aid to Families with Dependent Children), Housing assistance or Fuel assistance

**Fig. 1:**
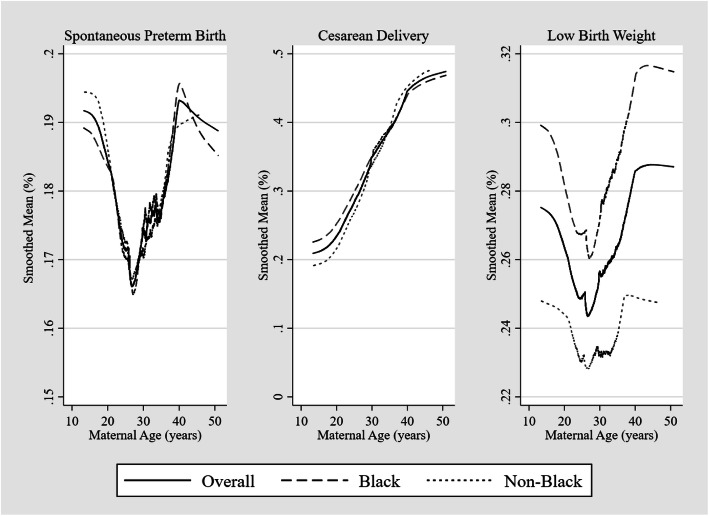
Lowess smoothing plots showing prevalence of adverse outcomes (spontaneous preterm birth, cesarean delivery, and low birth weight) by maternal age in total sample and stratified by racial groups

Adverse pregnancy outcomes among this population highlighted in Table 2 included spontaneous preterm birth (18%), cesarean delivery (33%) and low birth weight (26%). Spontaneous preterm delivery rates did not differ significantly across maternal age groups. Figure 1 highlights the distribution of adverse outcomes by maternal age and race. The relationship between both spontaneous preterm deliveries and low birth weight with age was sinusoidal with rates being highest among women less than 20 or more than 40 while cesarean deliveries increased linearly with age. Across all ages, low birth weight rates were higher among Blacks.

**Table 2:** Adverse outcomes in study population by maternal age groups (*N* = 8509)

***Adverse Pregnancy Outcomes***	< 20 years		20–29 years		30–39 years		40+ years		Total		***P***-value^**a**^
	N	%	N	%	N	%	N	%	N	%	
Total	907	100	4305	100	2960	100	337	100	8509	100	
*Spontaneous preterm delivery*											0.546
No	736	81.1	3561	82.7	2417	81.7	275	81.6	6989	82.1	
Yes	171	18.9	744	17.3	543	18.3	62	18.4	1520	17.9	
*Cesarean delivery (n = 8420)*											< 0.001
No	725	80.8	3045	71.6	1731	58.9	158	47.2	5659	67.2	
Yes	172	19.2	1206	28.4	1206	41.1	177	52.8	2761	32.8	
*Low birth weight*											0.037
No	659	72.7	3240	75.3	2147	72.5	242	71.8	6288	73.9	
Yes	248	27.3	1065	24.7	813	27.5	95	28.2	2221	26.1

**Fig. 2:**
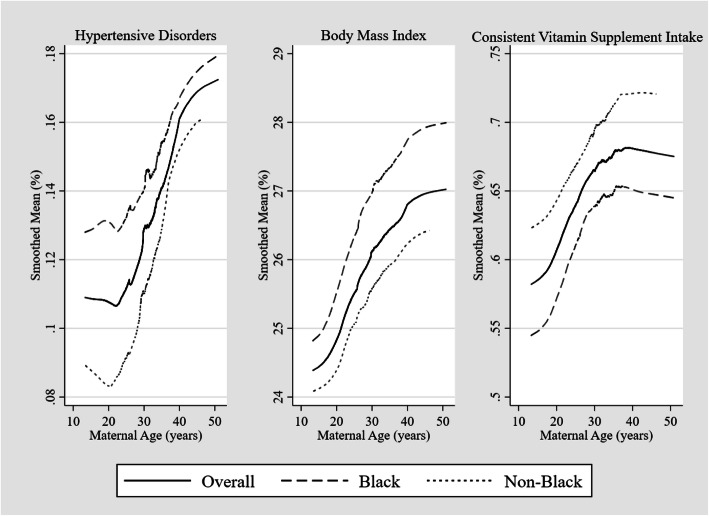
Lowess smoothing plots showing selected covariables (hypertensive disorders, BMI, and consistent multivitamin intake) by maternal age in total sample and stratified by racial groups

In Fig. 2, the distribution of some major risk factors of adverse outcomes as well as nutritional status by maternal age and race shows that the rates of hypertensive disorders and BMI increased with maternal age and was higher among Blacks. Consistent multivitamin supplement intake in pregnancy also increased with age, but was lower in Blacks .

Table 3 highlights the crude and adjusted odds of adverse outcomes across age groups (reference: 20–29 year olds), controlling for sociodemographic factors (maternal race, parity, education, marital status, receipt of public assistance, yearly income, nativity), biomedical conditions (obesity, hypertensive disorders and diabetes mellitus) and behavioral factors (including positive factors such as consistent intake of multivitamin supplements, support from father of baby, support from family, and negative factors such as major stress in pregnancy, cigarette smoking and alcohol intake).

**Table 3:** Crude and sequentially adjusted associations of maternal age with adverse pregnancy outcomes in all women (*N* = 8509) and among black women only (*N* = 4393)

Maternal Age	All Women		Black Women Only	
	Crude OR	Adjusted OR^**a**^	Crude OR	Adjusted OR^**a**^
**Spontaneous Preterm Birth**				
20–29 years (ref)	1.00	1.00	1.00	1.00
< 20 years	1.11 (0.92–1.34)	0.93 (0.76–1.13)	1.12 (0.87–1.45)	0.88 (0.67–1.16)
30–39 years	1.08 (0.95–1.21)	1.30 (1.14–1.49)	1.11 (0.94–1.32)	1.42 (1.18–1.71)
40+ years	1.08 (0.81–1.44)	1.39 (1.03–1.88)	1.02 (0.70–1.47)	1.37 (0.93–2.02)
**Cesarean Section**				
20–29 years (ref)	1.00	1.00	1.00	1.00
< 20 years	0.60 (0.50–0.72)	0.62 (0.51–0.74)	0.59 (0.46–0.75)	0.61 (0.47–0.79)
30–39 years	1.77 (1.60–1.95)	1.68 (1.51–1.87)	1.68 (1.47–1.93)	1.64 (1.41–1.92)
40+ years	2.84 (2.27–3.56)	2.58 (2.03–3.27)	2.52 (1.90–3.34)	2.42 (1.79–3.28)
**Low Birth Weight**				
20–29 years (ref)	1.00	1.00	1.00	1.00
< 20 years	1.14 (0.97–1.35)	0.92 (0.77–1.10)	1.17 (0.94–1.46)	0.91 (0.72–1.16)
30–39 years	1.15 (1.04–1.28)	1.36 (1.21–1.53)	1.19 (1.03–1.37)	1.58 (1.34–1.87)
40+ years	1.19 (0.93–1.53)	1.42 (1.09–1.85)	1.20 (0.88–1.62)	1.66 (1.19–2.32)

^a^ Covariates include: sociodemographic factors (maternal race, parity, education, marital status, receipt of public assistance, yearly income, nativity), biomedical conditions (obesity, hypertensive disorders and diabetes mellitus) and behavioral factors (including positive factors such as consistent intake of multivitamin supplements, support from father of baby, support from family, and negative factors such as major stress in pregnancy, cigarette smoking and alcohol intake)

Unadjusted models show no significant difference in spontaneous preterm birth odds by maternal age. However, adjusted odds were higher among women 30–39 year old (aOR: 1.30, 95% CI: 1.14, 1.49) and 40 years and older (aOR: 1.39, 95% CI: (1.03–1.88). Both crude and adjusted odds of cesarean section was significantly lower among women less than 20 years old (aOR: 0.62, 95% CI: 0.51, 0.74) but higher odds among women 30–39 (aOR: 1.68, 95% CI: 1.51, 1.87) and 40 years and older (aOR: 2.58, 95% CI: 2.03, 3.27). Similarly, both crude and adjusted odds of low birth weight was significantly higher among 30–39 (aOR: 1.36, 95% CI: 1.21, 1.53) and 40 years and older (aOR: 1.42, 95% CI: 1.09, 1.85). The trends in crude and adjusted odds of adverse outcomes persisted among a sub-sample of Black women only. Of note, the adjusted odds of spontaneous preterm birth (aOR: 1.42, 95% CI: 1.18, 1.71) and low birth weight (aOR: 1.58, 95% CI: 1.34, 1.87) were higher among 30–39 year old Black women compared to the full sample.

The changes in the odds ratios of adverse outcomes with sequential adjustments for sociodemographic, biomedical and behavioral factors are presented in Supplement Table 1 while further analysis on the influence of these factors on adverse outcomes is presented in Supplemental Table 2. Of note, Blacks had higher odds of low birth weight. While smoking and sub-optimal BMI worsened all outcomes, nulliparity was protective of all outcomes. Consistent multivitamin supplement intake, higher education and being married reduced spontaneous preterm birth and low birth weight but US nativity worsened all three outcomes.

**Fig. 3:**
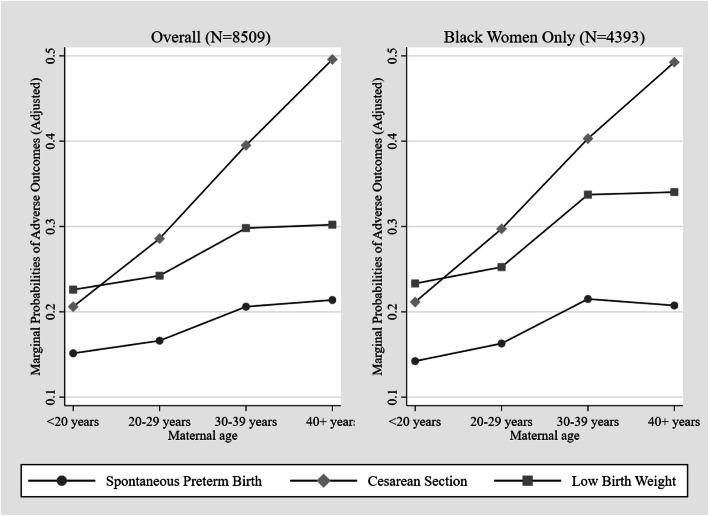
Marginal Probabilities of Adverse Outcomes after Adjusting for Sociodemographic, Biomedical, and Behavioral Factors by Maternal Age Overall and among Black Women only

Figure 3 highlights the marginal probabilities of adverse outcomes by maternal age after accounting for sociodemographic, biomedical, and behavioral factors. The marginal probability of spontaneous preterm birth increases gradually with maternal age from less than 20 years to 30–39 years with a very slight increase at 40 years or older. For cesarean section, the marginal probability increases linearly with maternal age from whereas for low birth weight, the marginal probability increases linearly for with maternal age up to 30–39 years old but does not change among women 40 years or older. The observed trends are unchanged for the sub-group of Black women only.

## Discussion

This study aimed to explore the relationship between maternal age and adverse outcomes- spontaneous preterm birth, cesarean delivery, and low birth weight - among a low-income minority US population. Our study shows that after accounting for race and other psychosocial risk factors, adverse pregnancy outcomes were higher among older mothers, particularly those 30 years or older. In addition, adverse outcomes were higher among Black women across the age spectrum. However, the relationship between maternal age and adverse outcomes varied depending on the sociodemographic, biomedical, and behavioral factors accounted for.

The increased risk of adverse outcomes with age may be due to an inherent increased risk in pregnancies in mothers at the extreme ages. Research suggests that adolescent pregnancy is associated with immature pelvises and an increased risk of longer labor and cesarean delivery indicated for failure to progress or descent [24]. However this study found adolescents at lower risk of cesarean delivery as well as hypertensive disorders. Aging typically results in a deterioration of most physiological functions [25], and chronological age is used as a marker for (though not equivalent to) biological or reproductive age. Increased cerebrovascular, cardiac, and respiratory morbidity in older mothers is partly attributed to physiological changes associated with aging, such as muscle atrophy, reduced cardiac reserve, atherosclerosis, and reduced lung function. Due to the added physiological burden of pregnancy, declining organ function may be more apparent in older women. Given the trend of increasing maternal age in the US, pregnancy complications are likely to persist or even rise, unless relevant public health and social interventions are instituted to optimize pregnancy outcomes among the growing population of older mothers.

This study reaffirms maternal age and race disparities in low birth weight demonstrated in the literature [26]. Racial disparities are typically explored without taking age groups into account, despite age being an important biologic factor for several outcomes. In contrast, this study explores disparities across the entire spectrum of reproductive age among a predominantly urban, low income (on public assistance) population. However, poor outcomes may be due to a constellation of causes beyond race/ethnicity, including health providers’ implicit/explicit bias, segregation, poverty, poorly-funded schools, etc., which disproportionately affect communities of color and manifest as low health literacy, poor access to high-quality care, lack of social support. To mitigate these disparities, a fundamental change in how care is provided to women, particularly low income Black women, may be needed. Women’s health care should seek to mitigate sociodemographic, biomedical and behavioral risk factors as well as employ a life course perspective in order to combat the observed persistent health disparities across the age spectrum. Our study highlighted potentially modifiable factors influencing adverse pregnancy outcomes. Such factors include optimal BMI, multivitamin supplement intake, level of education, social support, stress during pregnancy, and cigarette smoking. These findings underscore the importance of optimal nutritional status and social support in promoting healthy behavior across all age groups. The study suggests that maternal age, particularly adolescence (less than 20) and advanced maternal age (40 years or more) may be a social and public health issue given the influence of maternal age on individual health as well as population level outcomes [20, 27].

The study also observed that US nativity worsened the risk of adverse outcomes while nulliparity was protective. Our findings and other recent publication from the BBC [28, 29] corroborate the healthy migrant theory [30] which has been documented among South America and Asia born US migrants [31]. However, given this study does not disaggregate women by race and nativity, the degree to which this would apply to Black migrants specifically and warrants further investigation. The protective effect of nulliparity on adverse pregnancy has been well-documented and may be partly explained by its association with younger maternal age [32].

Study findings can inform tailored interventions in order to mitigate adverse health outcomes particularly among high risk groups such as Blacks or advanced maternal age pregnancies. Given the role of BMI and consistent vitamin intake on adverse outcomes, targeted interventions should start as early as possible and aim to improve nutritional status and may include promoting micronutrient supplementation/fortification, an individualized approach for food/nutrient provision, nutrition education, nutrition counselling and co-ordination of nutrition care across all ages with emphasis among Black mothers and those at the extreme ages, as well as those with a sub-optimal nutritional status, or low levels of social support [33].

Given the role of BMI on adverse outcomes, efforts to integrate individualized nutrition care into clinical guidelines for preconception and prenatal care should be thoroughly explored. A study demonstrated that an integrated WIC and obstetrical service model employing enhanced nutrition services and education is feasible and can limit postpartum weight retention in women with obesity [34]. Study findings reinforce the need for holistic interventions that take women’s context into account, employing the social ecological model which considers the complex interplay between individual, interpersonal, community, and societal factors influencing health. School based interventions [35] may prove beneficial for younger women while workplace interventions for older women [36]. Community level interventions should strive to create social support for pregnant women such as the use of in-person or virtual support groups where mothers with similar contexts can engage. Structural, and policy interventions are needed to improve availability of healthy food options particularly among low income populations that often live in food deserts. Furthermore, all women should have access to quality preconception, prenatal and postnatal health care, regardless of their age, race, or socioeconomic status. National and state level health insurance policy changes are critical and are urgently needed to ensure expanded access to health care particularly among low or middle income minority young women.

This study has a number of strengths. First, with a majority low income (on public assistance), racial minority, urban population, the BBC is representative of a high risk yet understudied population. Thus, study findings are generalizable to similar low income, minority, urban US populations. The study employed a rich dataset including sociodemographic, biomedical, and behavioral variables. In addition, the study explored associations with biological age groups- and did not include multiple gestations, major chromosomal abnormalities, congenital malformations, and IVF which typically occur more in older women.

However, the study acknowledges the following limitations. First, the data is cross sectional and cannot infer causality. Second, a number of outcomes including age, race and consistent multivitamin supplement were self-reported and this may potentially lead to misclassification. Of note, all adverse pregnancy outcomes and BMI status were objectively assessed using clinical data. The study does not explore other aspects of nutritional status such as diet and physical activity. In view of these, our findings warrant additional studies. In addition, due to the low prevalence of some racial groups such as Asians and AI/AN, this study was unable to fully explore some racial, ethnic or nativity differences that may explain some of the study findings.

In summary, our study found that in this predominantly urban, low-income, predominantly Black US population, adverse pregnancy outcomes differed significantly by maternal age. However, this relationship was complex and was in part attributable to confounding by sociodemographic, biomedical, and behavioral factors. Additional research and structural and policy interventions should take these factors into account.

## Supplementary Information

**Additional file 1 **: **Supplement Table 1.** Crude and Sequentially Adjusted Associations of Maternal Age with Adverse Pregnancy Outcomes (*N*=8509). **Supplement Table 2.** Maternal age, race, nutritional status, and other characteristics associated with adverse outcomes (*N*=8509).

## Data Availability

The datasets used and/or analyzed during the current study are available from the corresponding author on reasonable request.

## References

[1] Martin JA, Hamilton BE, Osterman MJ, Driscoll AK (2019). Births: final data for 2018.

[2] Mathews T, Hamilton BE (2002). Mean age of mother, 1970–2000. Natl Vital Stat Rep.

[3] Martin JA, Hamilton BE, Osterman MJ. Births: Provisional data for 2019. USA: Services USDoHaH, Prevention CfDCa, Statistics NCfH, System NVS; 2020.

[4] Duncan GJ, Lee KT, Rosales-Rueda M, Kalil A (2018). Maternal age and child development. Demography.

[5] Medicine PCotASfR (2014). Female age-related fertility decline. Committee opinion no. 589. Obstet Gynecol.

[6] Mathews TJ, Hamilton BE. Mean age of mothers is on the rise: United States, 2000-2014. USA: NCHS data brief. 2016(232):1–8. 26828319

[7] Lean SC, Derricott H, Jones RL, Heazell AE (2017). Advanced maternal age and adverse pregnancy outcomes: a systematic review and meta-analysis. PLoS One.

[8] Lisonkova S, Potts J, Muraca GM, Razaz N, Sabr Y, Chan W-S (2017). Maternal age and severe maternal morbidity: a population-based retrospective cohort study. PLoS Med.

[9] Callaghan WM, Creanga AA, Kuklina EV (2012). Severe maternal morbidity among delivery and postpartum hospitalizations in the United States. Obstet Gynecol.

[10] Azeez O, Kulkarni A, Kuklina EV, Kim SY, Cox S. Peer reviewed: hypertension and diabetes in non-pregnant women of reproductive age in the United States. Prev Chronic Dis. 2019;16. 10.5888/pcd16.190105. 10.5888/pcd16.190105 PMC6824149 31651378

[11] Campbell KH, Savitz D, Werner EF, Pettker CM, Goffman D, Chazotte C (2013). Maternal morbidity and risk of death at delivery hospitalization. Obstet Gynecol.

[12] Hinkle SN, Sharma AJ, Kim SY, Park S, Dalenius K, Brindley PL (2012). Prepregnancy obesity trends among low-income women, United States, 1999–2008. Matern Child Health J.

[13] Petersen EE, Davis NL, Goodman D, Cox S, Syverson C, Seed K (2019). Racial/ethnic disparities in pregnancy-related deaths—United States, 2007–2016. Morb Mortal Wkly Rep.

[14] Amjad S, MacDonald I, Chambers T, Osornio-Vargas A, Chandra S, Voaklander D (2019). Social determinants of health and adverse maternal and birth outcomes in adolescent pregnancies: a systematic review and meta-analysis. Paediatr Perinat Epidemiol.

[15] Margerison-Zilko CE, Li Y, Luo Z (2017). Economic conditions during pregnancy and adverse birth outcomes among singleton live births in the United States, 1990-2013. Am J Epidemiol.

[16] Biney AAE, Nyarko P (2017). Is a woman's first pregnancy outcome related to her years of schooling? An assessment of women's adolescent pregnancy outcomes and subsequent educational attainment in Ghana. Reprod Health.

[17] Kassa GM, Arowojolu AO, Odukogbe AA, Yalew AW (2019). Adverse neonatal outcomes of adolescent pregnancy in Northwest Ethiopia. PLoS One.

[18] Leftwich HK, Alves MV (2017). Adolescent Pregnancy. Pediatr Clin N Am.

[19] Narukhutrpichai P, Khrutmuang D, Chattrapiban T (2016). The obstetrics and neonatal outcomes of teenage pregnancy in Naresuan University Hospital. J Med Assoc Thail.

[20] Lemoine M-E, Ravitsky V (2015). Sleepwalking into infertility: the need for a public health approach toward advanced maternal age. Am J Bioeth.

[21] Malabarey OT, Balayla J, Klam SL, Shrim A, Abenhaim HA (2012). Pregnancies in young adolescent mothers: a population-based study on 37 million births. J Pediatr Adolesc Gynecol.

[22] Geronimus AT (2003). Damned if you do: culture, identity, privilege, and teenage childbearing in the United States. Soc Sci Med.

[23] Olapeju B, Saifuddin A, Wang G, Ji Y, Hong X, Raghavan R (2018). Maternal postpartum plasma folate status and preterm birth in a high-risk US population. Public Health Nutr.

[24] Ganchimeg T, Ota E, Morisaki N, Laopaiboon M, Lumbiganon P, Zhang J (2014). Pregnancy and childbirth outcomes among adolescent mothers: a World Health Organization multicountry study. BJOG Int J Obstet Gynaecol.

[25] Cohen W (2014). Does maternal age affect pregnancy outcome?. BJOG Int J Obstet Gynaecol.

[26] Berger BO, Wolfson C, Reid LD, Strobino DM (2021). Adverse birth outcomes among women of advanced maternal age with and without health conditions in Maryland. Womens Health Issues.

[27] Melnikas AJ, Romero D (2020). Ideal age at first birth and associated factors among young adults in greater new York City: findings from the social position and family formation study. J Fam Issues.

[28] Boakye E, Sharma G, Ogunwole SM, Zakaria S, Vaught AJ, Kwapong YA (2021). Relationship of preeclampsia with maternal place of birth and duration of residence among non-Hispanic black women in the United States. Circulation.

[29] Olapeju B, Ahmed S, Hong X, Wang G, Summers A, Cheng TL (2020). Maternal hypertensive disorders in pregnancy and postpartum plasma B vitamin and Homocysteine profiles in a high-risk multiethnic US Population. J Womens Health.

[30] Wingate MS, Alexander GR (2006). The healthy migrant theory: variations in pregnancy outcomes among US-born migrants. Soc Sci Med.

[31] Alexander GR, Mor JM, Kogan MD, Leland NL, Kieffer E (1996). Pregnancy outcomes of US-born and foreign-born Japanese Americans. Am J Public Health.

[32] Chan BC-P, Lao TT-H (2008). Effect of parity and advanced maternal age on obstetric outcome. Int J Gynecol Obstet.

[33] Salam RA, Hooda M, Das JK, Arshad A, Lassi ZS, Middleton P (2016). Interventions to improve adolescent nutrition: a systematic review and meta-analysis. J Adolesc Health.

[34] Gross SM, Augustyn M, Henderson JL, Baig K, Williams CA, Ajao B (2018). Integrating obstetrical care and WIC nutritional services to address maternal obesity and postpartum weight retention. Matern Child Health J.

[35] O'dea J (2000). School-based interventions to prevent eating problems: first do no harm. Eat Disord.

[36] Reed JL, Prince SA, Elliott CG, Mullen K-A, Tulloch HE, Hiremath S (2017). Impact of workplace physical activity interventions on physical activity and cardiometabolic health among working-age women: a systematic review and meta-analysis. Circulation.

