# Supplementary material for: Birth outcomes across the spectrum of maternal age: dissecting aging effect versus confounding by social and medical determinants
Source: BMC Pregnancy Childbirth. 2021 Sep 1;21:594. doi: 10.1186/s12884-021-04077-w (PMC8411515; doi:10.1186/s12884-021-04077-w)
Supplement: Supplementary file 1 — Additional file 1 : Supplement Table 1. Crude and Sequentially Adjusted Associations of Maternal Age with Adverse Pregnancy Outcomes (N=8509). Supplement Table 2. Maternal age, race, nutritional status, and other characteristics associated with adverse outcomes (N=8509). [file 12884_2021_4077_MOESM1_ESM.docx]

# Supplemental Materials

## Supplement Table 1: **Crude and Sequentially Adjusted Associations of Maternal Age with Adverse Pregnancy Outcomes** (N=8509)

| **Maternal Age** | **Model 1^1^** | **Model 2^2^** | **Model 3^3^:** | **Model 4^4^:** |
| --- | --- | --- | --- | --- |
|  | **Crude** | **Model 1 + sociodemographic** | **Model 2 + biomedical** | **Model 3 + behavior** |
| **Spontaneous Preterm Birth** | | | | |
| 20-29 years (ref) | 1.00 | 1.00 | 1.00 | 1.00 |
| <20 years | 1.11 (0.92 - 1.34) | 0.93 (0.77 - 1.13) | 0.91 (0.75 - 1.11) | 0.93 (0.76 - 1.13) |
| 30-39 years | 1.08 (0.95 - 1.21) | 1.24 (1.09 - 1.42) | 1.29 (1.13 - 1.47) | 1.30 (1.14 - 1.49) |
| 40+ years | 1.08 (0.81 - 1.44) | 1.32 (0.98 - 1.77) | 1.39 (1.03 - 1.88) | 1.39 (1.03 - 1.88) |
| **Cesarean Section** | | | | |
| 20-29 years (ref) | 1.00 | 1.00 | 1.00 | 1.00 |
| <20 years | 0.60 (0.50 - 0.72) | 0.56 (0.46 - 0.67) | 0.60 (0.49 - 0.72) | 0.62 (0.51 - 0.74) |
| 30-39 years | 1.77 (1.60 - 1.95) | 1.83 (1.65 - 2.04) | 1.70 (1.52 - 1.89) | 1.68 (1.51 - 1.87) |
| 40+ years | 2.84 (2.27 - 3.56) | 3.02 (2.40 - 3.79) | 2.59 (2.05 - 3.28) | 2.58 (2.03 - 3.27) |
| **Low Birth Weight** | | | | |
| 20-29 years (ref) | 1.00 | 1.00 | 1.00 | 1.00 |
| <20 years | 1.14 (0.97 - 1.35) | 0.85 (0.71 - 1.01) | 0.85 (0.72 - 1.02) | 0.92 (0.77 - 1.10) |
| 30-39 years | 1.15 (1.04 - 1.28) | 1.43 (1.27 - 1.60) | 1.37 (1.21 - 1.54) | 1.36 (1.21 - 1.53) |
| 40+ years | 1.19 (0.93 - 1.53) | 1.57 (1.21 - 2.02) | 1.43 (1.10 - 1.86) | 1.42 (1.09 - 1.85) |

^1^ covariates: maternal age

^2^ covariates: maternal age, race, parity, education, marital status, receipt of public assistance, nativity

^3^ covariates: maternal age, race, parity, education, marital status, receipt of public assistance, nativity, obesity, hypertensive disorders, diabetes mellitus

^4^ covariates: maternal age, race, parity, education, marital status, receipt of public assistance, nativity, obesity, hypertensive disorders, diabetes mellitus, consistent intake of multivitamin supplements, support from father of baby, support from family, major stress in pregnancy, cigarette smoking, alcohol intake

## Supplement Table 2: Maternal age, race, nutritional status, and other characteristics associated with adverse outcomes (N=8509)

| **Maternal characteristics** | **aOR (95% CI)^a^** | | |
| --- | --- | --- | --- |
|  | **Spontaneous Preterm Delivery** | **Cesarean Delivery** | **Low Birth Weight** |
| **Age** | | | |
| 20-29 years (ref) | 1.00 | 1.00 | 1.00 |
| <20 years | 0.91 (0.75 - 1.11) | 0.63 (0.52 - 0.76) | 0.87 (0.73 - 1.04) |
| 30-39 years | 1.27 (1.12 - 1.45) | 1.74 (1.56 - 1.94) | 1.45 (1.29 - 1.63) |
| 40+ years | 1.32 (0.98 - 1.77) | 2.82 (2.24 - 3.56) | 1.57 (1.21 - 2.03) |
| **Race** | | | |
| Non-Black (ref) | 1.00 | 1.00 | 1.00 |
| Black | 1.00 (0.90 - 1.13) | 1.04 (0.95 - 1.15) | 1.32 (1.19 - 1.46) |
| **Nutritional status** | | | |
| BMI |  |  |  |
| Normal weight (ref) | 1.00 | 1.00 | 1.00 |
| Underweight | 1.30 (1.04 - 1.62) | 0.82 (0.66 - 1.02) | 1.50 (1.24 - 1.83) |
| Overweight | 1.08 (0.95 - 1.24) | 1.32 (1.17 - 1.47) | 0.88 (0.78 - 1.00) |
| Obese | 0.84 (0.72 - 0.99) | 1.81 (1.60 - 2.05) | 0.90 (0.79 - 1.03) |
| Consistent multivitamin supplement intake in pregnancy | | | |
| No (ref) | 1.00 | 1.00 | 1.00 |
| Yes | 0.76 (0.67 - 0.85) | 1.03 (0.93 - 1.14) | 0.83 (0.74 - 0.92) |
| **Other characteristics** | | | |
| Nulliparous (ref: multiparous) | 0.90 (0.79 - 1.02) | 0.85 (0.77 - 0.95) | 0.75 (0.67 - 0.83) |
| College education  (ref: less than college) | 0.86 (0.76 - 0.97) | 1.07 (0.97 - 1.18) | 0.87 (0.78 - 0.97) |
| Married (ref: unmarried) | 0.87 (0.76 - 1.00) | 1.10 (0.99 - 1.22) | 0.84 (0.75 - 0.95) |
| Public assistance  (ref: no) | 0.95 (0.82 - 1.08) | 0.97 (0.86 - 1.09) | 0.99 (0.88 - 1.12) |
| US born (ref: no) | 1.40 (1.22 - 1.61) | 0.99 (0.88 - 1.11) | 1.42 (1.26 - 1.61) |
| Excellent father of baby support (ref: no) | 0.98 (0.85 - 1.13) | 0.90 (0.80 - 1.01) | 1.01 (0.89 - 1.15) |
| Excellent family support (ref: no) | 0.96 (0.83 - 1.10) | 1.11 (0.99 - 1.25) | 0.99 (0.88 - 1.12) |
| Major stress (ref: no) | 1.06 (0.93 - 1.22) | 1.13 (1.01 - 1.27) | 1.07 (0.95 - 1.21) |
| Smoke cigarettes (ref: no) | 1.38 (1.19 - 1.61) | 1.22 (1.06 - 1.40) | 1.69 (1.48 - 1.94) |
| Drink alcohol (ref: no) | 1.00 (0.82 - 1.21) | 1.03 (0.87 - 1.21) | 1.03 (0.87 - 1.22) |

^a^ Covariates include: sociodemographic factors (maternal race, education, parity, marital status, receipt of public assistance, yearly income, nativity), biomedical conditions (obesity, hypertensive disorders and diabetes mellitus) and behavioral factors (including positive factors such as consistent intake of multivitamin supplements, support from father of baby, support from family, and negative factors such as major stress in pregnancy, cigarette smoking and alcohol intake).
